# Development of Transcriptional Fusions to Assess *Leptospira interrogans* Promoter Activity

**DOI:** 10.1371/journal.pone.0017409

**Published:** 2011-03-18

**Authors:** Gustavo M. Cerqueira, Natalie M. Souza, Eduardo R. Araújo, Aline T. Barros, Zenaide M. Morais, Sílvio A. Vasconcellos, Ana L. T. O. Nascimento

**Affiliations:** 1 Centro de Biotecnologia, Instituto Butantan, São Paulo, Brazil; 2 Laboratório de Zoonoses Bacterianas do VPS, Faculdade de Medicina Veterinária e Zootecnia, São Paulo, Brazil; 3 Interunidades em Biotecnologia, Instituto de Ciências Biomédicas, São Paulo, Brazil; Tulane University, United States of America

## Abstract

**Background:**

Leptospirosis is a zoonotic infectious disease that affects both humans and animals. The existing genetic tools for *Leptospira* spp. have improved our understanding of the biology of this spirochete as well as the interaction of pathogenic leptospires with the mammalian host. However, new tools are necessary to provide novel and useful information to the field.

**Methodology and Principal Findings:**

A series of promoter-probe vectors carrying a reporter gene encoding green fluorescent protein (GFP) were constructed for use in *L. biflexa*. They were tested by constructing transcriptional fusions between the *lipL41*, Leptospiral Immunoglobulin-like A (*ligA*) and Sphingomielynase 2 (*sph2*) promoters from *L. interrogans* and the reporter gene. *ligA* and *sph2* promoters were the most active, in comparison to the *lipL41* promoter and the non-induced controls. The results obtained are in agreement with LigA expression from the *L. interrogans* Fiocruz L1-130 strain.

**Conclusions:**

The novel vectors facilitated the *in vitro* evaluation of *L. interrogans* promoter activity under defined growth conditions which simulate the mammalian host environment. The fluorescence and rt-PCR data obtained closely reflected transcriptional regulation of the promoters, thus demonstrating the suitability of these vectors for assessing promoter activity in *L. biflexa*.

## Introduction

*Leptospira interrogans* is the main causative agent of leptospirosis, a zoonotic infectious disease with worldwide distribution. Chronically infected reservoir hosts, such as rats, do not exhibit overt disease but are colonized by leptospires in their renal tubules and shed bacteria in the urine. Humans become infected by exposure to contaminated water, soil, or urine [1]. Severe manifestations of the disease, as observed in Weil's disease, are frequent and associated with significant mortality, up to 15% [1], [2]. In addition, leptospirosis may evolve to severe pulmonary haemorrhage syndrome (SPHS), for which case fatality is >50% [3], [4].

The *Leptospira* genus is composed of 20 recognized species and includes strains that belong to the saprophyte, intermediate or pathogen groups [5]. Currently, almost 300 serovars are recognized, of which more than 200 are considered to be pathogenic [2], [5], [6], [7]. The available genome sequences for pathogenic [8], [9], [10] and saprophytic [11] *Leptospira* spp. have been employed to search for new diagnostic reagents and vaccine candidates for leptospirosis.

Prompt diagnosis and early treatment of leptospirosis are essential to avoid severe outcomes [12]. The early phase leptospirosis is often misdiagnosed due to its presentation with nonspecific clinical signs [1], low sensitivity and frequent poor specificity of the results exhibited by the microscopic agglutination test (MAT) and the commercially available assays [7], [13], [14], [15], [16], [17], [18], [19].

The use of vaccines as prevention measures appears to be a cost-effective approach to prevent worldwide diseases. Commercially available whole-cell vaccines confer protection in a limited and incomplete manner, limiting their use among humans. E. g. whole-cell preparations produce only short-term immunity, requiring administration semi-annually; present low cross-protection and adverse reactions due to both residual media components and leptospiral lipopolysaccharide [2], [6], [7], [20], [21]. Efforts have been made for over a decade towards identifying immunoreactive [22], [23], [24], [25], [26], [27], [28] or protective [29], [30], [31], [32], [33], [34], [35], [36], [37], [38], [39], [40], [41] antigens via recombinant DNA technology. Despite these advances, limitations still remain to be overcome. The rational identification of novel candidate antigens is therefore necessary and the development of genetic tools to *Leptospira* spp. may be helpful for this purpose.

The mechanisms of *Leptospira* pathogenesis remain unclear, despite the efforts to identify virulence factors and their role in the pathogen-host interaction. To this aim, new genetic tools have been developed in the last years [11], [42], [43]. Functional characterization of outer membrane and cytoplasmic proteins [44], [45], [46], [47], [48], [49], and more recently the generation and study of knock-out mutants [50], [51], [52], [53], [54], [55], [56] have provided important contribution.

Many efforts have been done to understand the influence of environmental signals on the leptospiral transcriptome and proteome, aiming to identify antigens involved in pathogenesis [44], [46], [47], [48], [57], [58]. Temperature, physiological osmolarity, iron availability and the growth phase, in addition to the multitude of factors existing in the host serum and during pathogen-host cell contact, are known to affect the expression of several leptospiral proteins [57], [58], [59], [60], [61], [62]. pH was also found to be responsible for protein regulation, such as LipL36 and P31LipL45 (Qlp42), in the kidney tubules of hamsters [61], [63]. However, many leptospiral coding sequences (CDSs) still remain to be characterized [64]. The *L. interrogans* genome carries a considerably large number of genes supposedly involved in response regulation [9]. *L. borgpetersenii*, which possesses a reduced ability to survive outside the host, contains a lower number of regulatory genes [10]. However, the saprophyte *L. biflexa* contains a larger number of putative transcription factors than the other sequenced species. This suggests that *L. biflexa* can be used as a model to study the gene regulation of pathogenic *Leptospira* spp. [11], [57]. Despite the sequence diversity between both species, these findings suggest that pathogens and saprophytes might share some similar mechanisms to respond to the environment.

The extent to which the manipulation of *in vitro* conditions can be used to reproduce the full spectrum of mammalian host signals, which trigger differential gene expression in pathogenic *Leptospira* spp. remains uncertain. In this study, we sought to develop a new genetic tool to help elucidating the biology of *Leptospira* spp. We found that our promoter-probe methodology is useful for assessing promoter activity under defined conditions. The comparative analysis of the fluorescence produced by a specific *L. biflexa* reporter strain, PAG (carrying a copy of the *ligA* promoter fused to the *gfp* gene), and LigA expression from *L. interrogans* Fiocruz L1-130 strain, grown *in vitro* under the same systematic conditions, validated the use of *L. biflexa* as a model to assess *L. interrogans* promoter activity. The reporter strain containing a copy of the sphingomyelinase 2 promoter, *Psph2* (P2G) was also strongly induced by the conditions tested, above an established cut-off, whereas the non-virulence factor promoter control, from the *lipL41* lipoprotein gene, was not. We believe this promoter-probe methodology may support the existing methodologies to the identification of novel virulence factors of pathogenic *Leptospira* spp.

## Methods

### Bacterial strains and growth conditions

Bacterial strains and constructs are listed in Table 1. Strains were obtained from the collection of the Faculdade de Medicina Veterinária e Zootecnia, Universidade de São Paulo, São Paulo, Brazil; Laboratoire de Biologie des Spirochetes, Institut Pasteur, Paris, France and the ATCC. The virulence of the low-passage *L. interrogans* serovar Copenhageni Fiocruz L1-130 strain was maintained by passage through Golden Syrian hamsters. Low-passage refers to strains that were sub-cultured in EMJH liquid medium up to 10 times. All strains were cultured at 30°C in liquid EMJH medium supplemented with 1% rabbit serum [65], [66]. Chemically competent *E. coli* TOP10F cells were used as host for genetic manipulation of plasmids. *E. coli* transformants were typically selected on LB agar plates containing spectinomycin (50 µg/ml) or ampicillin (100 µg/ml). Electrocompetent *L. biflexa* sorovar Patoc strain Patoc 1 was prepared as previously described [55], and transformed with replicating shuttle vectors containing the promoter-probe cassettes (Table 1). EMJH plates were prepared using 1% agar and supplemented with spectinomycin at 50 µg/ml.

**Table 1:** Primers, plasmids and strains employed in this study.

Designation	Sequence (5′-3′)/genotype
**PRIMERS**	
PlipL41300F	CCGCTCGAGAGATAAGATCCAACCCAAAAGTTG
PlipL41300R	GGCGGATCCATGAAAAGTAACACCAATCCTGTTTGA
PligA300F	CCGCTCGAGTTGGTTTTATAGAAATCAGCAATGATCC
PligA300R	GGCGGATCCATAAACACTCACTCTAATTGTTTTATTTGAA
Psph2600F	CCGCTCGAGAAACAAAGAATACATACTATAACGTGAATTC
Psph2600R	GGCGGATCCATCGTTCTCTATCTCCATTCTGTATGTTTG
Gfp5	GTCGACGAGCTCGAGGGATCCATGAGTAAAGGAGAAGAA
Gfp3	TCAGATCTATTTGTGATGGTGATGGTGATGGTATAGTTCATCC
fD1	AGAGTTTGATCYTGGYTYAG
rP2	ACGGCTACCTTGTTACGACTT
NligA5	GCGGATCCTCCGTTACCGCAGCGGAACTTACTGAGAT
NligA3	CCCAAGCTTTTACCAGGCTCGATTACTTTT
**PLASMIDS**	
pGem-T easy	TA cloning vector; Amp^r^
pGem-T easy/*gfp*	pGem-T easy carrying a copy of the *gfp* gene
pGem-T easy/*PlipL41*/*gfp*	pGem-T easy/*gfp* carrying a copy of the *PlipL41* promoter upstream *gfp*
pGem-T easy/*PligA*/*gfp*	pGem-T easy/*gfp* carrying a copy of the *PligA* promoter upstream *gfp*
pGem-T easy/*P*sph2/*gfp*	pGem-T easy/*gfp* carrying a copy of the *Psph2* promoter upstream *gfp*
pSLe94	*E. coli*/*L. biflexa* shuttle vector; Spc^r^
pSLe94/*PlipL41*/*gfp*	*E. coli*/*L. biflexa* shuttle vector carrying the cassete *PlipL41* promoter-*gfp*
pSLe94/*PligA*/*gfp*	*E. coli*/*L. biflexa* shuttle vector carrying the cassete *PligA* promoter-*gfp*
pSLe94/*P*sph2/*gfp*	*E. coli*/*L. biflexa* shuttle vector carrying the cassete *Psph2* promoter-*gfp*
**STRAINS**	
*Escherichia coli* Top 10	F^−^ *mcrA* Δ(*mrr-hsdRMS-mcrBC*) Φ80*lacZ*Δ*M15* Δ*lacX74 recA1 ara* Δ*139* Δ(*ara-leu*)*7697 galU galK rpsL* (Str^r^) *endA1 nupG galK rpsL* (Str^r^) *endA1 nupG*
*L. biflexa* sv. Patoc str. Patoc1	Wild-type saprophytic strain
*L. interogans* sv. Copenhageni str. Fiocruz L1-130	Wild-type pathogenic strain
P41G	Str. Patoc1 carrying the pSLe94/*PlipL41*/*gfp* shuttle vector
PAG	Str. Patoc1 carrying the pSLe94/*PligA*/*gfp* shuttle vector
P2G	Str. Patoc1 carrying the pSLe94/*Psph2*/*gfp* shuttle vector

Bacterial cultures of the indicated strains (wt and knock-in mutants) were grown in LB medium overnight at 37°C with vigorous shaking (*E. coli*) or in EMJH medium at 30°C with moderate agitation (*Leptospira* spp).

Liquid EMJH pH 6.7 was prepared by adding concentrated HCl until the pH of choice was reached. Spermine (Sigma) was dissolved according to the manufacturer's instructions and supplemented in cultures at 200 µM. Physiologic osmolarity was induced by supplementation with 120 mM NaCl [44]. Before induction, cultures were grown at 30°C in EMJH until the late-exponential phase was reached (culture density of 10^8^ to 10^9^/ml). Growth was monitored by measuring the OD_420_ using an Ultrospec 2100 pro spectrophotometer (GE Healthcare). Cells were harvested (OD_420_ 0.5) and immediately stored at −20°C.

### Bioinformatics analyses

The sequences used for this study were obtained from the complete genome sequence of *L. interrogans* serovar Copenhageni strain Fiocruz L1-130 by using the SpiroScope (http://www.genoscope.cns.fr/agc/mage) database [67] and *Leptospira* Genome Project (http://aeg.lbi.ic.unicamp.br) database [9]. The Neural Network Promoter Prediction v.2.2 [68], TRES – Transcription Regulatory Element Search, which performs searches within the TRANSFAC database [69] and the PromScan program, that generates an alignment of known sequences and matrix frequency [70], were used to scan DNA sequences for potential binding sites. Repeats were identified by the EMBOSS programs available at http://bioweb.pasteur.fr/nucleic/intro-en.html#repeat.

### Construction of reporter vectors

The *gfp* gene [43] was amplified by PCR with Gfp5/Gfp3 primers pair, which introduced the SmaI, SacI, XhoI, BamHI, SmaI restriction sites. The resulting fragment was cloned into pGEM-T Easy (Promega) yielding the pGEM-T Easy/*gfp* construct. The *PlipL41*, *PligA* and *Psph2* promoters from *L. interrogans* serovar Copenhageni strain Fiocruz L1-130 were amplified by PCR using primers PlipL41300F/PlipL41300R, PligA300F/PligA300R and Psph2600F/Psph2600R, which introduced the *Xho*I and *BamH*I restriction sites, and cloned into pGEM-T Easy/*gfp* via the same sites. Promoter-probe vectors containing the *L. interrogans* promoters-*gfp* cassettes were constructed in the *E. coli*-*L. biflexa* pSLe94 shuttle-vector [71]. The cassettes were removed from pGEM-T Easy (Promega) by *Sma*I digestion and cloned in via *Pvu*II restriction site to give the new vectors, pSLe94/*PlipL41*/*gfp*, pSLe94/*PligA*/*gfp* and pSLe94/*Psph2*/*gfp* (Table 1). Electroporation of leptospires was performed as previously described [55], [71]. After 1 to 2 weeks of incubation, spectinomycin resistant transformants were used to inoculate liquid medium.

### Reporter gene assays

Cells for spectrofluorometry measurements were resuspended in 300 µl of deionized water and distributed 100 µl per well in a black 96-well OptiPlate-96F microplate (PerkinElmer). GFP fluorescence was measured using a 650-10 spectrofluorometer (PerkinElmer) at an excitation wavelength of 485 nm and an emission wavelength of 538 nm. The fluorescence intensity from samples was expressed as arbitrary fluorescence units, obtained at a wavelength of maximum emission. The mean specific activity from at least three independent assays is indicated in the results.

### Fluorescence microscopy

Qualitative expression of GFP was examined under UV light for detection of fluorescence. The cultures were induced and samples were collected at representative time-points, spun down and the resulting pellets were washed twice with 1× PBS to remove the culture medium. The pellets were then resuspended in 1× PBS, 20% Glycerol. Aliquots of 20 µl of the bacterial suspensions were applied to slides and sealed with a concentrated formaldehyde resin. Images of leptospires on cover slips were acquired using an IX81 inverted microscope (Olympus) equipped with 20× and 40× objectives and the CellR software (version 3.1). The UV filter sets used were DAPI (excitation: 350 nm – range is 330 to 380 nm, Emission: 460 nm) and DIC (transmitted light).

### RNA isolation and rt-PCR

Cells were harvested (OD_420_ 0.25) and RNA was isolated from bacteria with TRIzol reagent (Invitrogen), as described by the manufacturer. Then, isolated RNA (2 µg) was treated with DNaseI (Invitrogen), following manufacturer's instructions. cDNA was synthesized using SuperScript III First-strand (Invitrogen) according to the manufacturer's protocol. Samples were quantified and checked for purity using an Ultrospec 2100 pro spectrophotometer (GE healthcare) and agarose gel electrophoresis. The cDNA (representing 100 ng of RNA per reaction) was amplified with Taq DNA polymerase (Invitrogen) using primers pairs fD1/rP2 (control) [72], Gfp5/Gfp3 for reporter strains [43], or NligA5/NligA3, for *L. interrogans* serovar Copenhageni strain Fiocruz L1-130.

### Whole-cell ELISA

Whole-cell ELISA experiments were performed using a modification of previously described methods [45], [73]. Flat-bottom polystyrene high-binding microtitre plates (Corning) were coated overnight at 4°C with 100 ml per well of 10^8^ ml^−1^ whole *L. interrogans* serovar Copenhageni strain Fiocruz L1-130, which were previously centrifuged at 4,000×g and resuspended in 0.05 M sodium carbonate buffer (pH 9.6). Plates were blocked overnight at 4°C, and washed three times with 150 µl *Leptospira* Enrichment EMJH (Difco). Wells were incubated for 1 h at room temperature with 100 µl per well of a 1∶2,000 dilution of rabbit anti-LigA polyclonal serum (Kindly provided by Dr. Albert I. Ko and Dr. Paula Ristow, Gonçalo Moniz Research Centre, Oswaldo Cruz Foundation, Brazil) in *Leptospira* Enrichment EMJH, and washed three times with 200 µl PBS containing 0.05% Tween 20 (PBS-T). Wells were incubated with 100 µl per well of a 1∶5,000 dilution of horseradish-peroxidase-linked donkey whole-antibody anti-rabbit IgG (Sigma) for 1 h at room temperature, followed by two washes with 200 ml PBS-T, and three washes with PBS. The reactions were developed by adding 50 µl per well of *ο*-phenylenediamine (OPD) (1 mg/ml) in citrate phosphate buffer (pH 5.0) plus 1 µl/ml H_2_O_2_ was added (100 µl per well) for 10 min in the dark, at room temperature. The reaction was stopped by adding a 50 µl volume of 4 M H_2_SO_4_, and the absorbance was measured at 492 nm. Each ELISA experiment was repeated three times.

### Statistical analysis

Differences between average values were tested for significance by performing an unpaired, two-sided Student's t-test [74]. Differences were considered statistically significant when the resulting *p* values were ≤0.05.

## Results

### In vitro induction of virulent *Leptospira interrogans*

Many efforts have been made to identify and quantify the leptospiral transcriptome and proteome during growth under different conditions. But, so far, little is known about the influence that mammalian host conditions exert over promoter activity. Therefore, we initiated our investigation by studying *ligA* gene transcription and protein expression profiles using *L. interrogans* serovar Copenhageni strain Fiocruz L1-130 grown, for up to 24 h, under a combination of conditions that include the mammalian physiological osmolarity (∼300 mosmol–120 mM), temperature (37°C), urine pH (6.7) and the supplementation with spermine, a component belonging to the intracellular environment. We focused our study on the first 24 h of induction, to observe the changes that *L. interrogans* promoters undergo during the early stages of host invasion.

**Figure 1:**
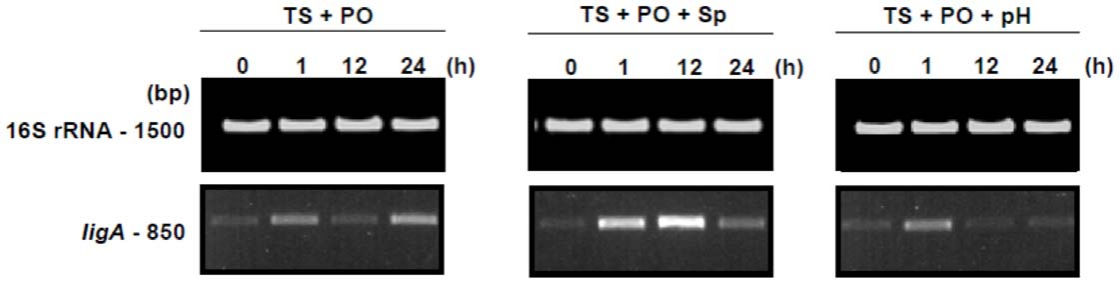
Influence of the *in vitro* conditions on native LigA expression by *L. interrogans* serovar Copenhageni str. Fiocruz L1-130. The effect of the various combinations of conditions (see Results section) on LigA expression was assessed by rt-PCR. Columns depict the systematic treatments: TS – Temperature upshift from 30°C to 37°C, PO – Physiological osmolarity, Sp – Spermine induction, pH – Urine pH induction. Within each gel the upper and lower bands correspond to the internal PCR control (16S rRNA) and *ligA* cDNA, respectively (domains 10–12). The lanes contain the amplified cDNA per sample time-point, both pre-treatment (0 h) and post induction (1, 12 and 24 h). Agarose gels were stained with GelRed (Invitrogen). No bands were observed in control samples run without template (data not shown). Samples were standardized according to an OD_420_ 0.25. Data from a representative significant study are shown.

Initially, we evaluated the influence of the different conditions by rt-PCR analysis (Figure 1). It was performed on RNA extracted from both non-induced and induced cultures of the virulent, low-passage, *L. interrogans* serovar Copenhageni strain Fiocruz L1-130. Low level transcription (*ligA*-based) was detected before induction of the virulent strain, while a clear difference in the abundance of *ligA* transcript could be seen after induction, and between the time intervals. Three time-points were evaluated (1, 12 and 24 h post induction – p.i.) and, interestingly, *ligA* transcription was not constant after induction (Figure 1). Under most conditions, the level of *ligA* transcript was higher than the non-induced control immediately one hour p.i. However, only spermine was able to up-regulate *ligA* for an extended period, up to 12 h p.i., while the other treatments exhibited down-regulation (Figure 1). At 24 h p.i., only the temperature upshift and physiological osmolarity conditions stimulated up-regulation in comparison to the previous time-point (Figure 1).

**Figure 2:**
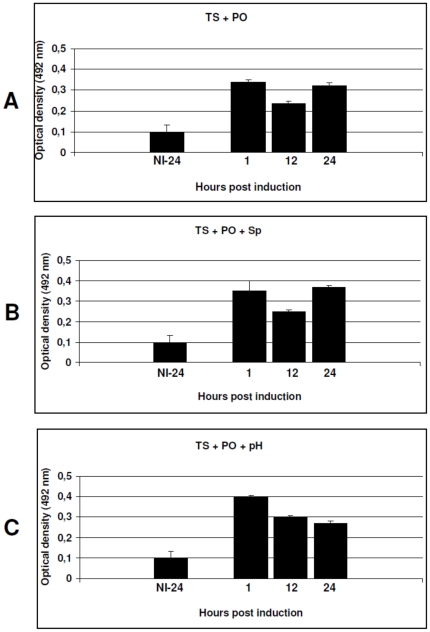
Native protein expression stimulated by the *in vitro* conditions mimicking those of the mammalian host. The expression levels of LigA were evaluated by ELISA, following exposure to the various conditions and combinations: TS – Temperature upshift from 30°C to 37°C, PO – Physiological osmolarity, Sp – Spermine induction, pH – Urine pH induction. (A) Temperature upshift and physiological osmolarity. (B) Temperature upshift, physiological osmolarity and spermine (intracellular level). (C) Temperature upshift, physiological osmolarity and pH reduction from 7.2 to 6.7. A non-induced *L. interrogans* serovar Copenhageni str. Fiocruz L1-130 (NI-24) was cultivated *in vitro* for 24 h under standard conditions as a control. Protein expression was measured at one, 12 and 24 h post-induction and the results are expressed as mean optical density ± standard error (bars). The assay was standardized according to an OD_420_ of 0.25. Data from two independent and significant experiments are shown.

To examine the effect of the conditions over the expression patterns of native LigA, we cultivated *L. interrogans* serovar Copenhageni strain Fiocruz L1-130 until late-exponential phase before induction. The combination of both temperature upshift and physiological osmolarity promoted induction of protein synthesis (Figure 2A). An up-regulation 4.98-fold higher than the non-induced control was observed at one hour p.i. (p<0.01). The influence of spermine was also evaluated in the kinetics assay and stimulated LigA expression above the control levels immediately after induction, one hour p.i. (5.95-fold, p<0.01), (Figure 2B). The urine pH condition was simulated by reducing the pH from 7.2 to 6.7 and the highest up-regulation was obtained by this treatment, one hour p.i. (5.92-fold, p<0.01), (Figure 2C). But it was the only condition to be followed by decreasing expression levels. The analysis of native LigA expression complemented the results obtained by rt-PCR, although correlation was not always observed between RNA and protein profiles (Figure 1 versus 2).

### Prediction of regulatory sequences by Bioinformatics

Previous studies demonstrated that upon induction by physiological osmolarity and temperature shift from 20°C to 37°C and 30°C to 37°C the *lipL41*, *ligA* and *sph2* genes are differentially expressed in *L. interrogans* serovar Copenhageni strain Fiocruz L1-130, *L. kirschneri* serovar Grippotyphosa strain RM52 and *L. interrogans* serovar Lai strain 56601 [44], [46], [47], [48]. The rational to select the *PlipL41*, *PligA* and *Psph2* promoters to compose and standardize our study was based on previous studies that characterized them extensively at the RNA and protein levels.

The *PlipL41*, *PligA* and *Psph2* promoters were predicted by bioinformatics based on the analysis of the *L. interrogans* serovar Copenhageni strain Fiocruz L1-130 genome sequence (AE016823). Different regions were identified, which are composed of direct and inverted repeats (data not shown).

To examine whether the computationally determined sequences comprise leptospiral regulatory elements, we fused the DNA segments to a promoterless *gfp* reporter. Both elements were subsequently cloned into an *E. coli*-*L. biflexa* shuttle vector depicted in Table 1, yielding the plasmids pSLe94/*PlipL41*/*gfp*, pSLe94/*PligA*/*gfp* and pSLe94/*Psph2*/*gfp* (Table 1). To ensure the accurate inclusion of the DNA sequences containing the regulatory elements in the study, we designed primers to amplify 300 bp upstream of the first ATG of *lipL41* and *ligA*, and 600 bp upstream of *sph2*. The larger length of the amplified fragment upstream of *sph2* was adopted to include possible distal elements that may play a role in the activity of the *Psph2* promoter, since the intergenic space to the next ORF, LIC12630 (hypothetical protein), is 650 bp. In the case of the *lipL41* and *ligA* genes the intergenic spaces are 114 bp and 456 bp, respectively. The plasmid constructs were used to transform *L. biflexa* serovar Patoc strain Patoc I and generated the reporter strains P41G (*lipL41* promoter), PAG (*ligA* promoter) and P2G (*sph2* promoter), which were induced for production of fluorescence by the green fluorescent protein (GFP).

### Application of promoter-probe vectors in *L. biflexa*

A series of replicating promoter-probe vectors utilizing *gfp* were constructed to examine changes in promoter activity in response to a set of defined conditions (Table 1). The constructs were based on the low-copy number shuttle-vector pSLe94 (Table 1), which contains the LE1 origin of replication that is capable of replication within saprophytic *Leptospira* spp. To validate the usefulness of these vectors as genetic tools to investigate promoter regulation, transcriptional fusions were constructed including the promoters from the well-characterized genes *lipL41*, *ligA* and *sph2* of *L. interrogans* serovar Copenhageni strain L1-130. GFP reporter strains were established by electroporation of the promoter-specific reporter vectors into *L. biflexa*. The effectiveness of the reporter strains for assaying transcriptional activity was assessed quantitatively.

**Figure 3:**
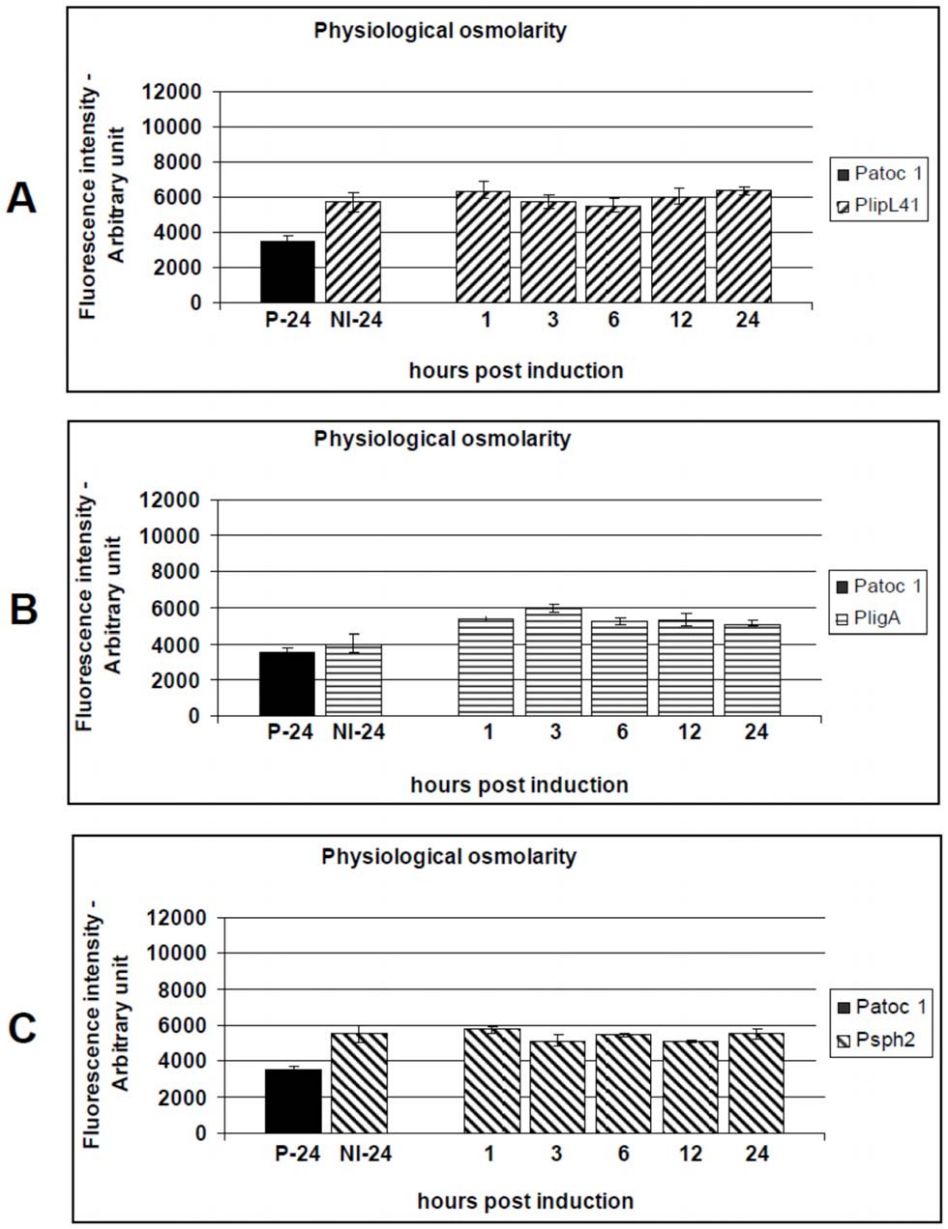
Kinetics of GFP production by the *L. biflexa* reporter strains (P41G, PAG and P2G) following induction by NaCl to a physiological level. Cultures of the reporter strains containing a single-copy transcriptional reporter and either the wild-type *lipL41* (A), *ligA* (B) or *sph2* (C) promoter regions were induced with ∼300 mosmol NaCl (physiological level). An induced non-transformed *L. biflexa* serovar Patoc str. Patoc 1 (P-24) was induced for 24 h as a control. The uninduced reporter strains (NI-24) were included as additional controls. Transcriptional activity was presented as the mean ± standard error (bars). Fluorescence levels from triplicate samples of each culture were standardized according to an OD_420_ 0.5 and are expressed as arbitrary fluorescence units. Data from a representative significant study are shown.

All three reporter strains of *L. biflexa* were initially cultivated on EMJH agar plates, at 30°C, and then transferred to liquid EMJH, both containing low-salt concentration (70 mosmol per liter NaCl – 28 mM). When cultures reached the late-exponential phase they were aliquoted and either adjusted to 120 mM NaCl or temperature-upshfited from 30°C to 37°C. Quantitative assessment of fluorescence showed that, under physiological osmolarity conditions, the highest promoter activity was detected in the PAG reporter strain, compared to the control, p<0.01 (Figure 3). The *PligA* promoter was induced in the presence of 120 mM NaCl and the fluorescence reached the highest level 3 hours p.i. The average intensity at this time-point was 1.48-fold higher than the non-induced control (Figure 3B). Neither the *PlipL41* nor *Psph2* promoters were significantly induced (Figure 3A and 3C). Of note, there were no obvious differences between the growth curves of the wild-type and the reporter strains of *L. biflexa*, suggesting that the GFP expression did not cause any disadvantage to the host strain (data not shown).

**Figure 4 pone-0017409-g004:**
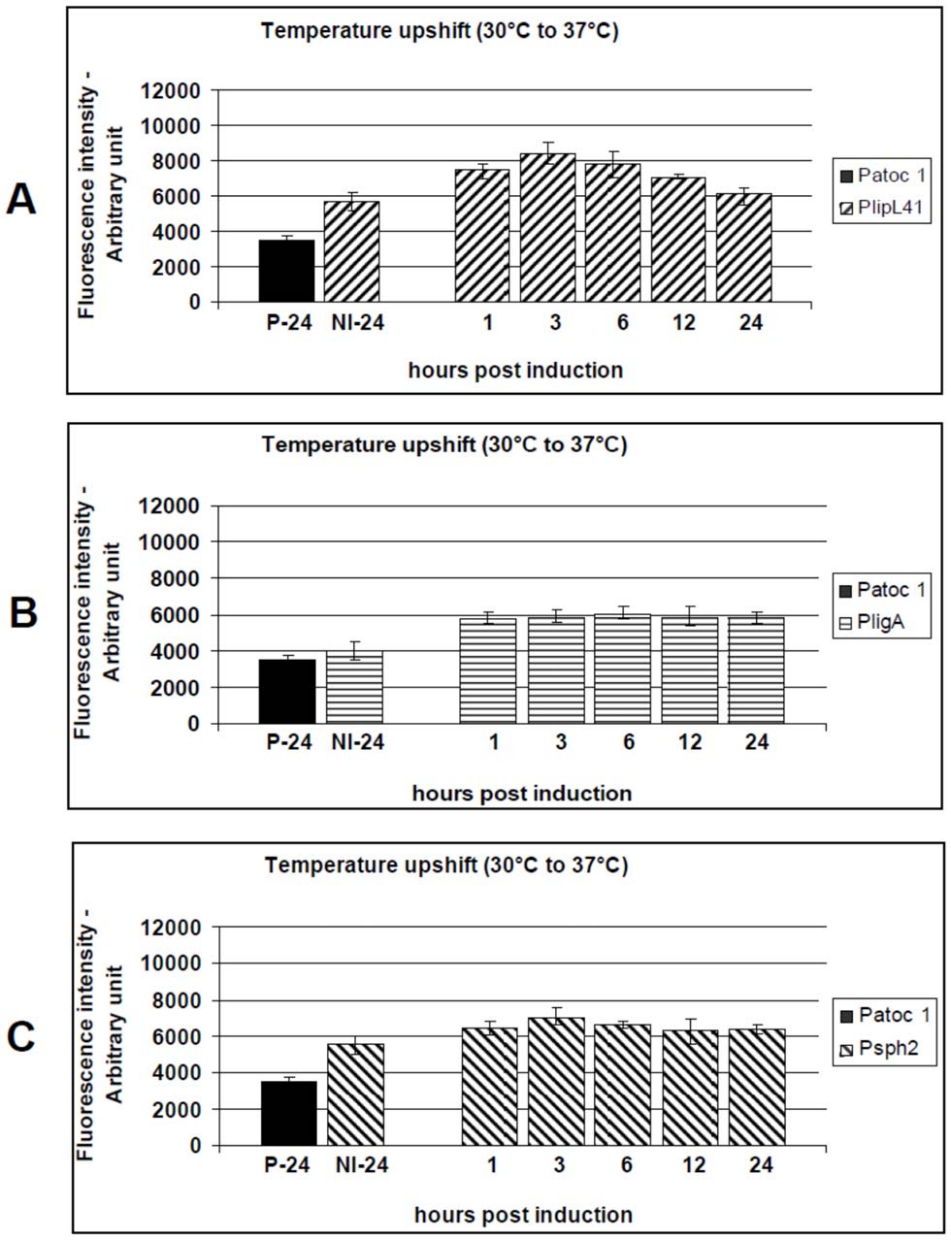
Kinetics of GFP production by the *L. biflexa* reporter strains (P41G, PAG and P2G) after a temperature upshift to 37°C. Cultures of the reporter strains P41G (A), PAG (B) or P2G (C) were induced by temperature upshift from 30°C to 37°C (physiological temperature). *L. biflexa* serovar Patoc str. Patoc 1 (P-24) induced for 24 h and uninduced reporter strains grown (NI-24) were included as controls. Transcriptional activity was measured for 24 h and presented as the mean ± standard error (bars). Fluorescence from triplicate samples of each culture were standardized according to an OD_420_ 0.5 and are expressed as arbitrary fluorescence units. Data from a representative significant study are shown.

The influence of the temperature upshift, from 30°C to 37°C, on promoter activity was also assessed. The levels of fluorescence produced by the P41G, PAG and P2G reporter strains were significantly higher than the non-induced controls (p<0.01). The *PlipL41* and *Psph2* promoters reached their maximum activity 3 hours p.i. (Figure 4A and 4C). At this time point there was significant (p<0.01) up-regulation of 1.46-fold and 1.26-fold, respectively. The P41G reporter strain exhibited the highest promoter activity, under this condition, followed by P2G. *PligA* promoter activity was most influenced, with expression 1.50-fold higher than the non-induced control, p<0.01. Interestingly, *PligA* and *Psph2* activities remained constant during the assay conditions (Figure 4B and 4C). The induction of *PlipL41* and *Psph2* by the temperature upshift led to production of fluorescence levels significantly higher than those induced under conditions of physiological osmolarity, p<0.05 (Figure 3A versus 4A and 3C versus 4C), while the *PligA* activity induced by the temperature upshift did not differ from that observed by salt supplementation, at most of the time-points investigated (Figure 3B versus 4B).

### Effect of the combination of environmental signals on *L. interrogans* promoter activity

**Figure 5 pone-0017409-g005:**
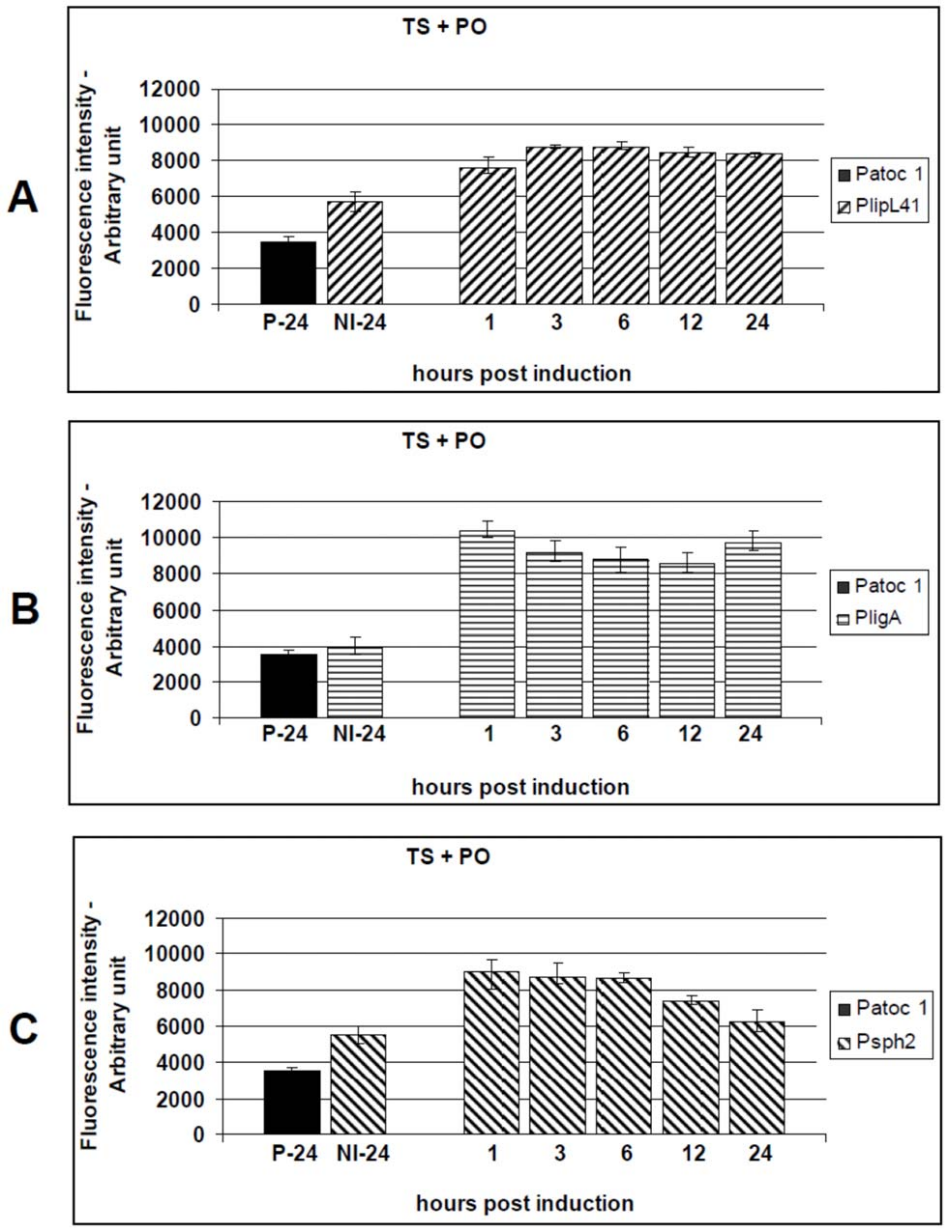
Kinetics of GFP production by the *L. biflexa* reporter strains (P41G, PAG and P2G) after exposure to a combination of physiological salt levels and temperature upshift to 37°C. Cultures of the reporter strains P41G (A), PAG (B) or P2G (C) were stimulated by a combination of physiological osmolarity (PO) and temperature upshift (TS). The induced wild-type strain (P-24) and the uninduced reporter strains (NI-24) were included as controls. Transcriptional activity was measured for 24 h and presented as the mean ± standard error (bars). Fluorescence from triplicate samples of each culture were standardized according to an OD_420_ 0.5 and are expressed as arbitrary fluorescent units. Data from a representative significant study are shown.

To identify promoters that are differentially induced in response to a combination of conditions, we performed systematic experiments with different, overlapping, stimuli. Subcultures were initially induced under conditions of physiological osmolarity and normal human body core temperature (37°C). The average fluorescence intensity produced by the reporter strains was highly significant when compared to the non-induced controls, p<0.01. For P41G, the highest promoter activity was observed 3 hours p.i. (1.54-fold), and remained constant throughout the assay (Figure 5A). Both *PligA* and *Psph2* were induced to the highest levels 1 hour p.i., 2.59-fold and 1.62, respectively (p<0.01). However, they did not maintain a constant activity during the assay (Figure 5B and 5C). Additionally, *PlipL41* was induced to a similar extent by the temperature upshift and the combination of physiological osmolarity and temperature (Figure 4A versus 5A). *PligA* and *Psph2* were significantly induced, 1.74-fold and 1.57-fold, respectively (Figure 3B versus 5B and 3C versus 5C), and 1.73-fold and 1.28-fold, respectively (Figure 4B versus 5B and 4C versus 5C), compared to the single-condition treatments (physiological osmolarity or temperature upshift versus their combination).

**Figure 6 pone-0017409-g006:**
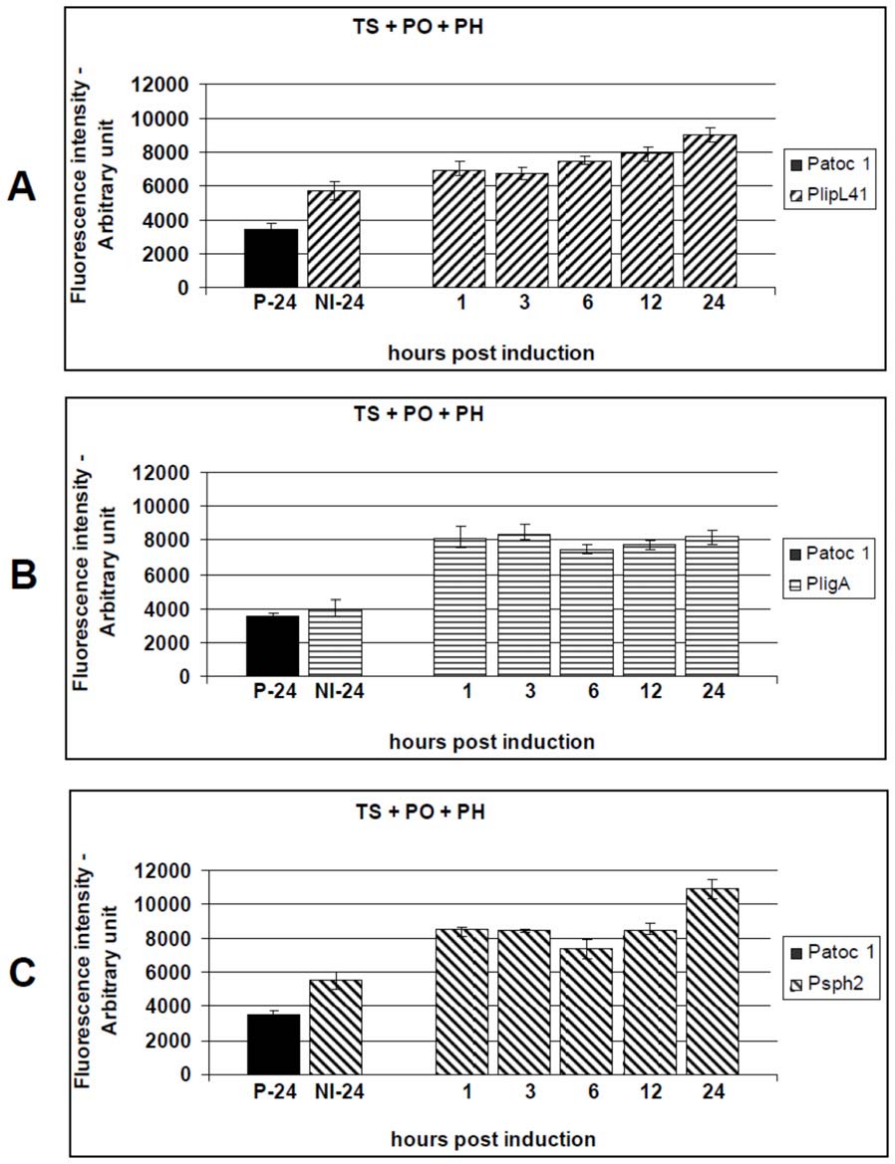
Kinetics of GFP production by the *L. biflexa* reporter strains (P41G, PAG and P2G), after exposure to a combination of physiological salt levels, temperature upshift to 37°C and urine pH 6.7. Cultures of the reporter strains P41G (A), PAG (B) or P2G (C) were induced by a combination of physiological osmolarity (PO), temperature upshift (TS) and pH reduction from 7.2 to 6.7 (PH). The induced wild-type control (P-24) and the uninduced reporter strains (NI-24) were included as controls. Transcriptional activity was measured for 24 h and presented as the mean ± standard error (bars). Fluorescence from triplicate samples of each culture were standardized according to an OD_420_ 0.5 and are expressed as arbitrary fluorescent units. Data from a representative significant study are shown.

The results indicate these promoters were preferentially active, *in vitro*, when *L. biflexa* was cultured under conditions emulating the mammalian host environment. Thus, we decided to evaluate another combination of conditions, the effect of the urine pH plus the physiological osmolarity and the temperature upshift. The reporter strains were sub-cultured into EMJH medium supplemented with 120 mM NaCl, pH 6.7, and shifted from 30°C to 37°C. This resulted in the activation of all promoters as seen by increasing levels of fluorescence. The results from three independent experiments are shown in Figure 6. The *PligA* promoter was the most affected, reaching an activity 2.08-fold higher than the non-induced control (3 hours p.i.), followed by *Psph2* (1.96-fold) and *PLipL41* (1.58-fold), both at 24 hours p.i, p<0.01 (Figure 6). Curiously, we observed that both *PligA* and *PlipL41* were slightly less stimulated by the combination that included pH 6.7 than that including salt and temperature only (p<0.05) (Figure 5A versus 6A and 5B versus 6B).

**Figure 7:**
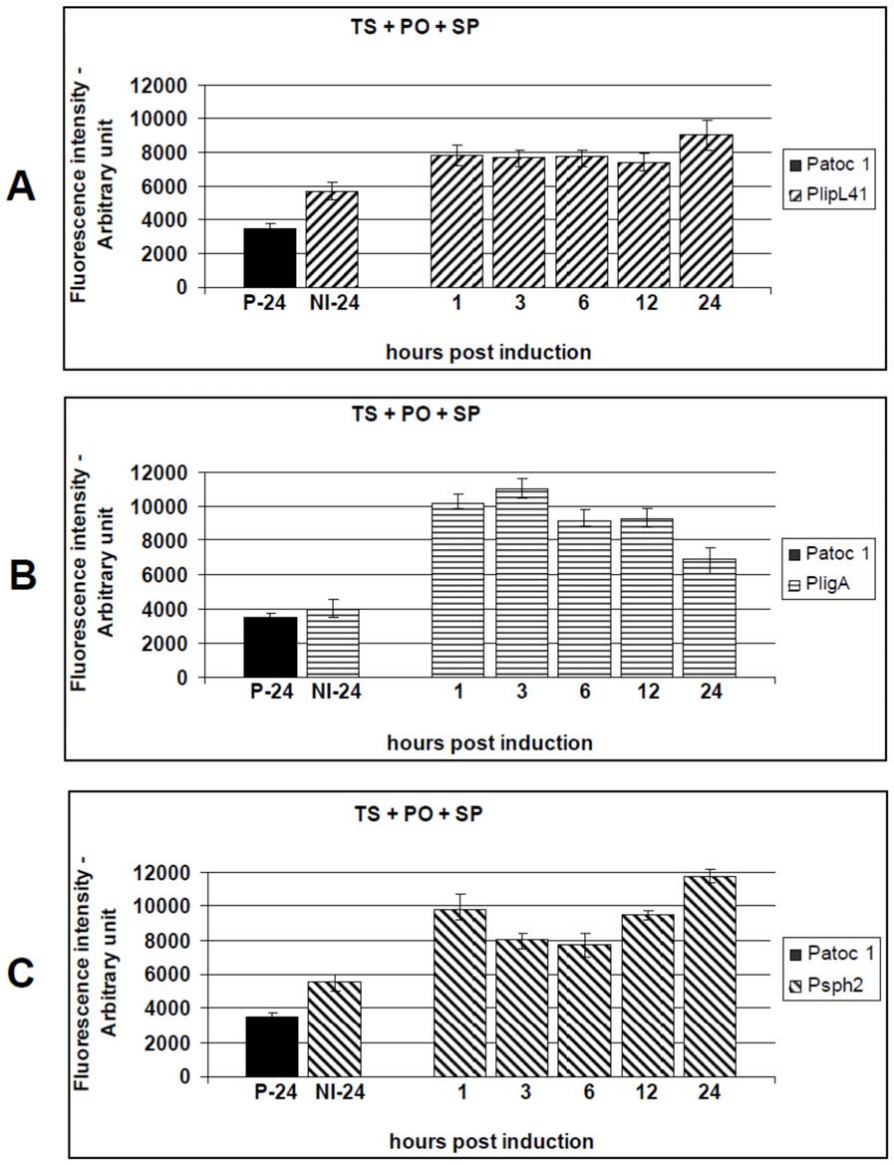
Kinetics of GFP production by the *L. biflexa* reporter strains (P41G, PAG and P2G), after exposure to a combination of physiological salt levels, temperature upshift to 37°C and supplementation with spermine. Cultures of the reporter strains P41G (A), PAG (B) or P2G (C) were induced by a combination of physiological osmolarity (PO), temperature upshift (TS) and 200 µM spermine (intracellular level) (SP). The induced wild-type control (P-24) and the uninduced reporter strains (NI-24) were included as controls. Transcriptional activity was measured for 24 h and presented as the mean ± standard error (bars). Fluorescence from triplicate samples of each culture were standardized according to an OD_420_ 0.5 and are expressed as arbitrary fluorescent units. Data from a representative significant study are shown.

Previous studies have demonstrated that several bacteria undergo translational regulation in response to spermine [75]. This polyamine is only produced by eukaryotic cells, it can reach millimolar levels within them [76], and it has significant effects on various cellular processes in liver, kidney and brain cells and in lymphocytes [76], [77], [78], [79], [80], [81], [82]. Here, we investigated whether spermine contributed to *L. interrogans* promoter regulation. The *L. biflexa* reporter strains were grown at 30°C until late-exponential phase and then supplemented with 200 µM spermine, 120 mM NaCl and upshifted to 37°C. The *ligA* promoter was the most affected, the PAG reporter strain produced a fluorescence level 2.74-fold higher than the non-induced control (3 hours p.i.), followed by P2G (2.12-fold) and P41G (1.58-fold), both 24 hours p.i, p<0.01 (Figure 7). However, *ligA* promoter activity was not maintained, the average fluorescence decreased to 1.72-fold (24 hours p.i.), demonstrating that *PligA* is transiently induced by spermine (Figure 7B). The spermine-induced activity of both *PligA* and *Psph2* promoters was the highest observed in this study (Figure 7B and 7C).

In agreement with the native LigA expression pattern, the fluorescence produced by the PAG reporter strain reached the highest (4.98-fold versus 2.59-fold) and lowest (2.19-fold versus 2.13-fold) levels at one and 12 hours p.i., respectively, when induced by the combination of physiological osmolarity and temperature upshift (Figure 2A versus 5B and Table 2), while the LigA expression pattern induced by spermine was different from the fluorescence intensity produced by the PAG reporter strain (Figure 2B versus 7B). Finally, induction by pH 6.7 led to LigA expression that was consistent with the fluorescence levels produced by the PAG reporter strain (Figure 2C versus 6B and Table 2). Protein and fluorescence levels were highest one hour p.i., but the amount of native LigA decreased significantly (from 5.95-fold to 4.04-fold), while the fluorescence level produced by the PAG reporter strain remained constant (from 2.07-fold to 2.03-fold). The results obtained in this study, by the use of the promoter-probe methodology described, allowed us to establish a cut-off based on the determined fluorescence levels (Table 2). This information will be useful to support the future prediction of virulence factors as we are currently developing a follow-up study employing several other promoter sequences to construct a library of knock-in mutants.

**Table 2:** Comparison among top fluorescence intensities and protein expression levels reached by the strains in study.

Treatments	Reporter strain		
	P41G	PAG	P2G
Physiological osmolarity	1.11	1.48	1.03
Temperature upshift to 37°C	1.46	1.50	1.26
Physiological osmolarity/Temperature upshift	1.54	2.59*	1.62
Physiological osmolarity/Temperature upshift/pH 6.7	1.58	2.08*	1.96†
Physiological osmolarity/Temperature upshift/Spermine	1.58	2.74*	2.12*

	Native protein		
		LigA	
Native protein - Physiological osmolarity/Temperature upshift	NA	4.98* ‡	NA
Native protein - Physiological osmolarity/Temperature upshift/pH 6.7	NA	5.92* ‡	NA
Native protein - Physiological osmolarity/Temperature upshift/Spermine	NA	5.95* ‡	NA

*Corresponds to signal intensity at least 2-fold higher than that observed for the non-induced control.

† Corresponds to the border line of 2-fold higher signal intensity, compared to the non-induced control.

NA - Not applicable.

‡ Relative to the parental virulent strain, *L. interrogans* serovar Copenhageni str. Fiocruz L1-130 strain.

**Figure 8:**
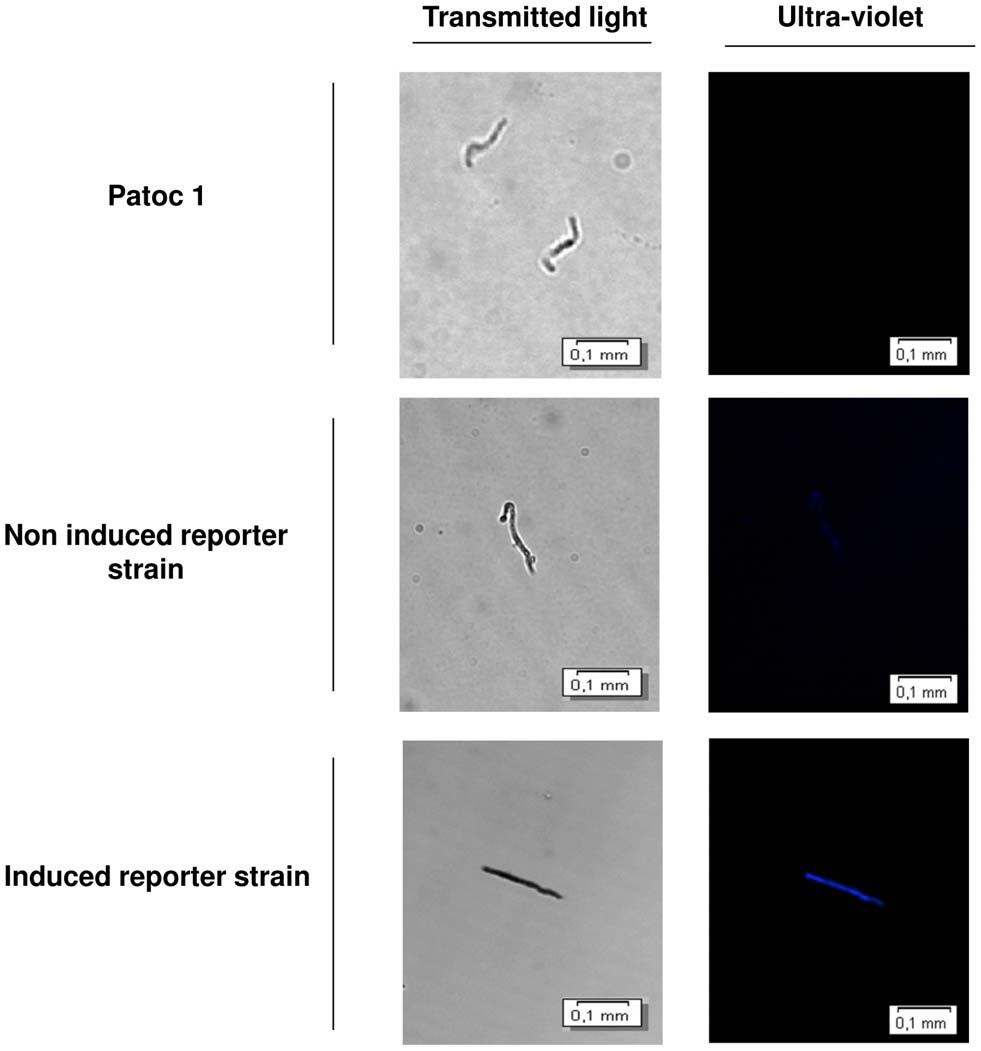
Inducible expression of GFP in the *L. biflexa* promoter-probe constructs. Microscope images of the *in vitro* induced and uninduced P2G reporter strain transformed with pSLe94-*sph2* promoter-*gfp* vector. Uninduced and physiological osmolarity/temperature upshift/spermine exposed leptospires were fixed and visualized by phase contrast and by GFP fluorescence. Wild-type *L. biflexa* str. Patoc 1 was used as the negative control.

The ability of the promoter-probe system to allow for the differentiation between both non-induced and induced reporter strains suggests that the *gfp* regulation can be employed to accurately mimic the behaviour of the native genes. This is corroborated by similarities observed between the expression patterns of native LigA and the fluorescence levels produced by the PAG reporter strain, in comparison to the non-induced controls (Table 2). Qualitative assessment of these reporter strains clearly demonstrated the presence of fluorescent leptospires upon induction *in vitro* (Figure 8).

**Figure 9:**
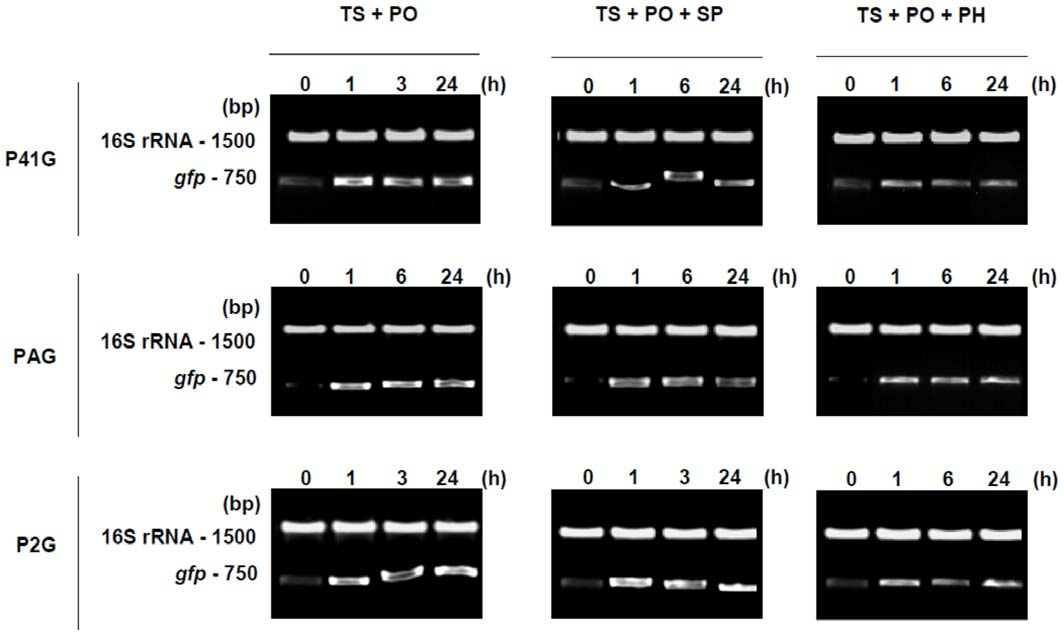
Influence of the *in vitro* conditions on *gfp* expression of the reporter strains. The effect of each of the combinations of conditions on promoter activity was assessed by the level of *gfp* mRNA produced. Rows depict the reporter strains in this study, while columns show the various conditions used: TS – Temperature upshift from 30°C to 37°C; PO – Physiological osmolarity; SP – Spermine induction; PH – Urine pH induction. Within each lane, the upper band represents the internal PCR control (16S rRNA) and the lower band corresponds to the *gfp* cDNA amplified by rt-PCR. The lanes in each gel contain the amplified cDNA per time-point, both pretreatment (0 h) and post induction (1, 3 or 6 and 24 h). Agarose gels were stained with GelRed (Invitrogen). No bands were observed in control samples run without template (data not shown). Samples were standardized according to an OD_420_ 0.25. Data from a representative significant study are shown.

To further substantiate the data obtained by the use of the promoter-probe vectors, rt-PCR analysis was performed on RNA extracted from non-induced and induced cultures of the *L. biflexa* reporter strains (Figure 9). As expected, low level of transcripts (*gfp*-based) were detected before induction of the reporter strains. Three time-points were evaluated, 1, 3 or 6 and 24 h p.i., but only a slight difference was observed between the time-points for any given *in vitro* treatment, per reporter strain (Figure 9). Despite this, the amount of *gfp* mRNA varied considerably per treatment. Correlation between the fluorescence and transcription levels was not always evident, similarly to the lack of correlation observed during the analysis of the native strain (see Results). In both cases (native and reporter strains) the discrepancy observed between the mRNA and protein levels probably resulted from intrinsic regulatory mechanisms. Although we have no information on the signalling pathways involved, the *L. interrogans* promoters evaluated appear to behave similarly in both species, suggesting conservation of the regulatory mechanisms higher than expected. The transcription data complement those obtained by fluorescence measurement from the reporter strains, reinforcing the utility of the promoter-probe vectors as a genetic tool to test promoter activity in *L. biflexa*.

Validation of the *L. biflexa* promoter-probe constructs was performed by comparison against the *in vitro*-induced pathogenic strain. In addition to the protein content, we compared the mRNA profiles produced by the wild-type virulent *L. interrogans* and the PAG reporter strain. Side-by-side analysis of two time-points (1 and 24 h p.i.) showed that all treatments produced similar patterns of both LigA and GFP expression, the latter being controlled by the native *L. interrogans PligA* promoter (Figure 1 versus 9). However, a noticeable difference was observed with respect to the transcript levels produced by the native *ligA* and the *PligA*-controlled *gfp* (Figure 1 versus 9). Of note, up-regulation of the *PligA* was noted despite the treatment (Table 2), therefore validating the use of this promoter-probe methodology as a genetic tool to assess the ability of pathogens promoters to respond host stimuli.

## Discussion

In this study we developed and characterized in detail a *L. biflexa* promoter-probe methodology for the analysis of *L. interrogans* promoters. The three promoters regions used in this study resemble regulatory regions of other bacterial species, based on bioinformatics characterization only (data not shown). Although we did not support *in silico* findings with *in vitro* data we observed that *PlipL41* promoter sequence is similar to the *E. coli* σ70 promoter, while *PligA* and *Psph2* organization is more distant to it. The identification of different repeats into the promoter regions studied may be suggestive of specific response regulator binding sites. It is possible that the elements through which each promoter senses the changes on environmental cues may be different.

The *lipL41*, *ligA* and *sph2* genes are known to be regulated *in vitro* by physiological conditions [44], [46], [47], [48]. In addition, the *L. interrogans* transcriptome was evaluated in response to multifactorial conditions, upon exposure to serum [58]. To further substantiate the knowledge of *Leptospira* genetics, P41G, PAG and P2G reporter strains were constructed to contain *gfp* under the conditional control of the aforementioned *L. interrogans* promoters. Analysis of cell extracts harvested from exponentially growing cultures revealed low GFP activity under routine growth conditions, while variable activity was observed post-induction. In agreement to previous findings obtained by transcriptomics, our data demonstrate the sensitivity of leptospiral promoters under the various conditions evaluated.

*ligA* is a paralog of *ligB*, and originated by duplication events of the first ten immunoglobulin-like domains [83], consequently both are expected to respond to host stimuli through similar mechanisms. Both proteins seem to contribute to the pathogenesis of *Leptospira* spp. as important adhesins [84], [85], [86]. In addition, spectral counts of both proteins by a mass-spectrometry-based strategy revealed that both LigA and LigB are present in 553 and 914 copies per cell, respectively [87]. Recently, Lo and colleagues were not able to detect LigB among *L. interrogans* serovar Lai samples shifted from environmental to human body temperature (37°C), demonstrating that temperature upshift alone may be insufficient for induction of expression of Lig proteins [88]. Likewise, in our study, the *PligA* promoter activity was only weakly detected during non-induced and single-condition induced PAG cells. However, fluorescence was more than doubled after induction by combinations of stimuli (Figure 5B–[Fig pone-0017409-g006] 7B). The same was observed for the expression of native LigA by *L. interrogans* serovar Copenhageni strain Fiocruz L1-130 (Figure 2).

Sph2 is expressed under routine *in vitro* conditions and temperature upshift to 37°C [87], [88], [89]. At the mRNA level, it is up-regulated by physiological osmolarity [47] and temperature [46]. In our study, we corroborated these findings with the observation of low levels of fluorescence in the non-induced P2G reporter strain and elevated levels of *Psph2* promoter activity after induction by the combination of different mammalian host conditions (Figures 5C, 6, 7C). These findings suggest the mammalian host conditions may have contributed to this effect and reinforces the hypothesis that the *sph2* gene is likely to be regulated at post-transcriptional level.

Interestingly, we observed LigA down-regulation when *L. interrogans* serovar Copenhageni was cultivated at pH 6.7 (Figure 2C). This is in agreement with previous findings that leptospiral antigens are down-regulated when leptospires are excreted in the urine [90]. Of note, this was not observed during fluorescence analysis (Figure 6). This suggests that specific response regulators may be triggered in the parental pathogenic strain, during renal colonization and/or urine shedding. In general, the activity of the *PlipL41* promoter was not, or only weakly, influenced under the various conditions studied (Figure 3A–[Fig pone-0017409-g004] [Fig pone-0017409-g005] [Fig pone-0017409-g006] 7A), and this is consistent with previous observations [59], [88], [91], [92]. In contrast to the study of Nally and colleagues [93] that observed LipL41 downregulation upon experimental infection, *PlipL41* promoter activity was not reduced when the P41G reporter strain was cultivated under the majority of the conditions analysed (Figure 3A–[Fig pone-0017409-g004] [Fig pone-0017409-g005] [Fig pone-0017409-g006] 7A). This suggests that the reduction of LipL41 expression in guinea pig-recovered *L. interrogans* may be due to post translational regulation, and may involve complex regulation systems such as host cell contact, protein degradation, and energy metabolism.

Of note, the *PlipL41* promoter was initially selected as a constitutive promoter. However, we noted that this promoter was also up-regulated at some of the selected time-points. An ideal constitutive gene should exhibit constant activity independently to the cell state or environmental conditions. However, constitutive expression may not be related to constant promoter activity. Moreover, expression levels of constitutive genes such as the flagellin or the ribosomal RNA synthesis genes, have been shown to be altered by temperature-shift or ciprofloxacin supplementation [87], [88]. *PlipL41* promoter activity was not always induced under the *in vitro* conditions. In addition, its activity was below the cut-off level (2-fold) established in this work for virulence factor promoters (Table 2). This lead us to believe that the P41G reporter strain may serve as a negative control in the identification of novel virulence factors.

Although *L. interrogans* is a pathogen known to infect the mammalian host through active penetration, there is uncertainty over the existence of an intracellular phase during this process. In this study, we demonstrated that spermine, a polyamine found in abundance in eukaryotic cells [76] alters *L. interrogans* promoters activity. Previous studies showed leptospiral survival and replication within human macrophages, a spermine-rich eukaryotic cell [94]. We observed that supplementation of reporter strain cultures with spermine was able to stimulate promoter activity. In addition, we present evidence that similar transcriptional and translational changes occurred during induction of the native proteins, supporting the hypothesis that pathogenic *Leptospira* spp. can recognize polyamines as a signal of the intracellular environment. Polyamine recognition is likely a component of a sophisticated system that integrates multiple environmental signals and regulates gene expression in intracellular bacteria, i.e. *Francisella* [95], [96], [97], and biofilm formation in *Vibrio* spp. and *Yersinia* spp. [77], [78]. As polyamines in the intracellular environment are likely to reach elevated levels, our findings on promoter regulation by spermine may be relevant bacterial pathogenesis in general.

It is not known what controls *ligA* or *sph2* transcription initiation. However, previous studies have shown that both genes are up-regulated by physiological osmolarity [44], [47], [48] and temperature [46]. Also, recent work presented evidence of a functional redundancy between LigA and LigB [52]. The finding that the *PligA* promoter is active under different *in vitro* conditions, which simulate the mammalian host environment, raises the hypothesis that *ligA* absence from several pathogenic serovars is likely to be due to spontaneous deleterious events that may have occurred inside or outside the mammalian host environment [83], [98], without any influence on virulence or pathogenicity. Since most of the conditions studied induced constant activity of *PligA in vitro* (Figure 3B–[Fig pone-0017409-g004] [Fig pone-0017409-g005] 6B), it is possible that LigA may contribute to the early stages in the course of leptospirosis. The kinetics of native LigA expression also supports this hypothesis, although protein levels did vary (Figure 2).

We found that native LigA was up-regulated upon induction by the combined conditions of physiological osmolarity, temperature upshift to 37°C and spermine, with a 5.95-fold increase. The corresponding reporter strain, PAG, was up-regulated by the same treatment (2.74-fold), demonstrating a good correlation between the promoter-probe methodology and native protein expression in *L. interrogans*, suggesting that LigA is post-transcriptionally regulated (Table 2). This also suggests that *L. interrogans* protein expression may involve particular mechanisms or pathways to respond to specific host stimuli. The overall up-regulation of native LigA observed in comparison to the non-induced control was in agreement with the induction of the *L. biflexa* PAG reporter strain. Our results are in concordance with previous studies whereby the *lig* genes were expressed *in vitro* at both transcript and proteins levels [99], although a previous study using an *L. interrogans* serovar Pomona type Kennewicki strain was only able to detect the *lig* genes at the transcript level, [100]. We believe that these differences may be due to the different strains used. Moreover, *ligB* a paralogous gene to *ligA*, was up-regulated by an overnight temperature upshift from 30°C to 37°C (1.7-fold). Yet, physiological osmolarity stimulated *ligA* and *ligB* up-regulation (4.41 to 5.27-fold – based on the transcriptional signals employing oligonucleotides to both the *ligA*/*ligB* identical region and the *ligB* unique region, respectively) [46], [48]. Additionally, a recent study evaluated the influence of serum on *ligB* up-regulation (1.89-fold) [58]. Although we did not quantify the transcription of the *ligA* gene, a clear variation was observed in comparison to the non-induced controls, which is in agreement with previous findings (Figure 1). This reinforces the applicability of the promoter-probe vectors to assess promoter activity in *L. biflexa*. Based on these results, we conclude that (*i*) the combination of the *in vitro* conditions reliably simulated the host environment, (*ii*) *L. biflexa* can be used as a model to characterize *L. interrogans* promoters and (*iii*) this promoter-probe methodology may be helpful in the prediction of potential virulence factors of pathogenic *Leptospira* spp. Furthermore, previous studies showed *ligA* gene up-regulation shortly after temperature upshift [46], [88], thus reinforcing the hypothesis that LigA may contribute to the early stages of infection and host adaptation. In addition, the *Psph2* promoter was up-regulated in the presence of spermine (2.12-fold).

We observed some conflicting evidence in the correlation between native *ligA* mRNA and LigA abundance levels (Figure 1 versus 2), similar to previous studies [88], [101], [102], [103], [104]. As previously suggested by Lo and colleagues [88], it is possible that the lack of correlation might be due to the longer half-life, greater stability or post transcriptional regulation of the mRNA transcript, i.e. as a result of the activity of small non-coding RNAs. Native LigA was detected at very low levels in the non-induced *L. interrogans* cells, while a significant up-regulation, at similar rates to recent studies [48], was observed when leptospires were grown under mammalian host conditions. In light of this information, we may conclude that the expression of both LigA and Sph2 is likely to be regulated at the post-transcriptional level.

Despite advances in the development of genetic tools for *Leptospira* spp. some basic questions remain unanswered. It is still unclear what regulators and pathways are associated with the expression of virulence factors. The level at which members of a given regulatory cascade exert induction/repression of transcription of LigA, Sph2 and other virulence factors is unknown. In this sudy we demonstrate that *L. biflexa* can serve as a model to study the genetics of *L. interrogans*. The pathogen-derived promoters exhibited activity in the *L. biflexa*, suggesting the existence of shared regulatory mechanisms among the saprophytes and pathogenic *Leptospira* spp. In addition, the establishment of a cut-off based on the promoter activity of virulence and non-virulence factors (Table 2), and the different fluorescence levels expressed by the reporter strains upon induction eliminated the possibility that the effects were simply due to stress responses. Furthermore, the suite of promoter-probe vectors developed in this study can be modified to make diverse translational fusions to investigate protein expression patterns and complex regulatory networks involved in leptospiral protein regulation.

In conclusion, this study demonstrates the potential of a novel genetic tool for the identification and characterization of virulence factors of *Leptospira* spp. Our transcription and expression findings suggest that a combination of *in vitro* signals may be important to accurately simulate the host environment. We expect to provide further information towards understanding *Leptospira* spp. genetics and that will eventually serve as a basis for further studies. The understanding of the extent to which leptospiral promoters are regulated by mammalian host conditions, as well as the expression kinetics, may reveal useful information about the biology of *Leptospira* spp. A knock-in mutant library of different promoters is currently under construction with this in mind.
